# Efficacy of low molecular weight heparin for chronic obstructive pulmonary disease and respiratory failure: A protocol of systematic review of randomized controlled trials

**DOI:** 10.1097/MD.0000000000018051

**Published:** 2019-11-27

**Authors:** Dejun Zhao, Jun-Fei Feng

**Affiliations:** a Department of Respiratory Medicine, People's Hospital of Fuyang, Hangzhou; b Department of Respiratory Medicine, Hangzhou Fuyang Hospital of Traditional Chinese Medicine, Hangzhou, China.

**Keywords:** low molecular weight heparin, chronic obstructive pulmonary disease, respiratory failure, randomized controlled trial, efficacy, safety

## Abstract

**Background:**

Evaluating the efficacy and safety of low molecular weight heparin (LMWH) for patients with chronic obstructive pulmonary disease (COPD) and respiratory failure (RF) is a major purpose of this study.

**Methods:**

The following electronic databases will be comprehensively retrieved from the inception to July 1, 2019: Cochrane Library, PUBMED, EMBASE, Google Scholar, Web of Science, Allied and Complementary Medicine Database, WANGFANG, and China National Knowledge Infrastructure without language restrictions. All randomized controlled trials related to LMWH for COPD and RF will be included. Two authors will carry out study selection, data collection, and risk of bias assessment independently.

**Results:**

This study will systematically explore the efficacy and safety of LMWH for COPD and RF. The primary outcome is lung function. The secondary outcomes are severity of dyspnea on exertion, quality of life, body mass index, airflow obstruction; and any expected and unexpected adverse events.

**Conclusion:**

The findings of this study will provide evidence to judge whether LMWH is an effective treatment for patients with COPD and RF.

**PROSPERO registration number:**

PROSPERO CRD42019 139631.

## 1 Introduction

Chronic obstructive pulmonary disease (COPD) is one of the most common lung diseases in the clinical practice.^[1,2]^ It also has been estimated as the third leading cause of death.^[3,4]^ It is characterized by airflow limitation and chronic airway inflammation.^[5–8]^ Patients with such condition often experiences increase in cough, sputum production, and dyspnea.^[9,10]^ If COPD can not been managed effectively and timely, it can cause respiratory failure (RF) in patients with such disorder.^[11–15]^

Previous studies have reported that low molecular weight heparin (LMWH) can be used to treat patients with COPD and RF.^[16–21]^ However, no systematic study has been conducted to investigate the efficacy and safety of patients with COPD and RF. Thus, this study will explore the efficacy and safety of LMWH for the treatment of patients with COPD and RF.

## 2 Methods and analysis

### 2.1 Ethics and dissemination

This study dose not needs ethical approval because no individual data will be used. We expect to publish the results of this study at a peer-reviewed journal.

### 2.2 Study inclusion and exclusion criteria

#### 2.2.1 Types of studies

All randomized controlled trials (RCTs) of using LMWH for patients with COPD and RF will be included with no language limitation.

#### 2.2.2 Types of interventions

Experimental intervention must include LMWH in any forms.

Comparison intervention can be any treatments except any forms of LMWH.

#### 2.2.3 Types of participants

Patients with COPD and RF will be considered for inclusion in spite of race, sex, age, education background, and economic status.

#### 2.2.4 Types of outcome measurements

Primary outcome includes lung function, which can be measured by forced expiratory volume in 1 second or any other related tools.

Secondary outcomes consist of severity of dyspnea on exertion, as measured by any relevant scales, such as the 6-minute walk test scale; quality of life, as assessed by St. George's Respiratory Questionnaire or other tools; body mass index; airflow obstruction; and any expected and unexpected adverse events.

### 2.3 Search methods for the identification of studies

#### 2.3.1 Electronic database searches

The following electronic databases will be searched comprehensively from inception to July 1, 2019: Cochrane Library, PUBMED, EMBASE, Google Scholar, Web of Science, Allied and Complementary Medicine Database, WANGFANG, and China National Knowledge Infrastructure without language restrictions. All RCTs related to the LMWH for COPD and RF will be considered with no language limitation. The detailed search strategy for Cochrane Library is presented in Table 1. We will also adapt similar retrieval strategy to the other electronic databases.

**Table 1:**
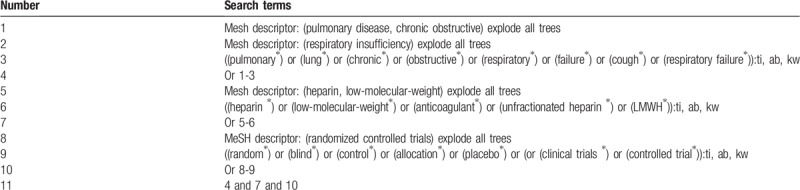
Search strategy for Cochrane Library.

#### 2.3.2 Other literature sources search

The reference lists of included RCTs and conference proceedings will be searched to avoid missing any eligible studies.

### 2.4 Data collection and analysis

#### 2.4.1 Selection of studies

Titles and abstracts of all searched records will be screened by 2 independent authors, focusing on identifying eligible studies and excluding duplicated or irrelevant studies according to the eligibility criteria. Full texts of possible trials will be further obtained if necessary. Any disagreements between 2 authors will be solved by a third author via discussion. The study selection is showed in the flowchart.

#### 2.4.2 Data extraction and management

Before data extraction, we will create a standard data extraction sheet including the following information: title, first author, year of publication, location, participant characteristics, eligibility criteria, sample size, study setting, study methods, randomization, blinding, treatment details, controls, outcomes, safety, conflicts of interest, and other information. All data collection will be extracted by 2 independent authors. A third author will be involved to solve any disagreements between 2 authors by discussion. Any insufficient or missing information will be inquired by contacting primary authors.

### 2.5 Study quality assessment

Two authors will independently assess the risk of bias for all included studies using Cochrane risk of bias tool. Seven domains of this tool will be evaluated, and each one will be further divided into 3 levels: low, unclear and high risk of bias. A third author will be set to solve all disagreements between 2 authors.

### 2.6 Treatment effect measurements

Continuous variables will be presented as mean difference or standard mean difference and 95% confidence intervals, while dichotomous values will be exerted as risk ratio and 95% confidence intervals.

### 2.7 Statistical analysis

We will utilize RevMan 5.3 software to carry out statistical analysis. *I*^*2*^ test is used to investigate heterogeneity among included studies. *I*^*2*^ ≤ 50% indicates having acceptable heterogeneity, and we will use a fixed-effects model. We will also conduct meta-analysis if it is possible. On the other hand, *I*^*2*^ > 50% means having significant heterogeneity, and we will use a random-effects model. In addition, subgroup analysis will be carried out to investigate the possible causes. If there is still substantial heterogeneity after subgroup analysis, descriptive summary will be reported.

### 2.8 Additional analysis

#### 2.8.1 Subgroup analysis

Subgroup analysis will be performed if the data are sufficient based on the different treatments, controls and outcomes.

#### 2.8.2 Sensitivity analysis

Sensitivity analysis will be carried out to assess the impact of study design, and methodological quality.

#### 2.8.3 Reporting bias

We will identify any reporting bias among eligible studies using funnel plot and Egger regression test if included studies are sufficient.^[22,23]^

## 3 Discussion

Although a variety of studies have shown that LMWH is effective in treating patients with COPD and RF, the evidence of LMWH in relieving COPD and RF is still insufficient. In order to systematically investigate the efficacy and safety of LMWH on all aspects of COPD and RF treatment, the goal of this study is to include adequate researches to ensure sufficient evidence. We expect LMWH to have a more positive impact on patients with COPD and RF. This result of this study may help provide more reliable evidence for the enhancement of LMWH management and application in the treatment of COPD and RF.

## Author contributions

**Conceptualization:** De-jun Zhao, Jun-fei Feng.

**Data curation:** De-jun Zhao, Jun-fei Feng.

**Funding acquisition:** De-jun Zhao.

**Investigation:** De-jun Zhao.

**Methodology:** De-jun Zhao, Jun-fei Feng.

**Project administration:** De-jun Zhao.

**Resources:** Jun-fei Feng.

**Software:** De-jun Zhao, Jun-fei Feng.

**Supervision:** De-jun Zhao.

**Validation:** De-jun Zhao, Jun-fei Feng.

**Visualization:** De-jun Zhao, Jun-fei Feng.

**Writing – original draft:** De-jun Zhao, Jun-fei Feng.

**Writing – review & editing:** De-jun Zhao, Jun-fei Feng.
